# 5-Amino-3-eth­oxy-1,8,8-trimethyl-2-aza­bicyclo­[2.2.2]octa-2,5-diene-4,6-dicarbo­nitrile

**DOI:** 10.1107/S1600536812032990

**Published:** 2012-08-01

**Authors:** Suchada Chantrapromma, Thitipone Suwunwong, Pumsak Ruanwas, Nawong Boonnak, Hoong-Kun Fun

**Affiliations:** a Crystal Materials Research Unit, Department of Chemistry, Faculty of Science, Prince of Songkla University, Hat-Yai, Songkhla 90112, Thailand; b Department of Chemistry and Center of Excellence for Innovation in Chemistry, Faculty of Science, Prince of Songkla University, Hat-Yai, Songkhla 90112, Thailand; c X-ray Crystallography Unit, School of Physics, Universiti Sains Malaysia, 11800 USM, Penang, Malaysia

## Abstract

The title 2-aza­bicyclo­[2.2.2]octa-2,5-diene derivative, C_14_H_18_N_4_O, crystallized out with two independent mol­ecules with similar conformations in the asymmetric unit. In each mol­ecule, the three six-membered rings adopt boat conformations. The mol­ecules exist in the enamine form. In the crystal, mol­ecules are linked by N—H⋯O and N—H⋯N hydrogen bonds into a two-dimensional network parallel to the *ab* plane.

## Related literature

For bond-length data, see: Allen *et al.* (1987 ▶). For ring conformations, see: Cremer & Pople (1975 ▶). For a related structure, see: Nakano *et al.* (1987 ▶). For background to 2-aza­bicyclo­[2.2.2]octa-2,5-diene derivatives, see: Igarashi *et al.* (1987 ▶); Nakano *et al.* (1999 ▶). For the stability of the temperature controller used in the data collection, see: Cosier & Glazer (1986 ▶).
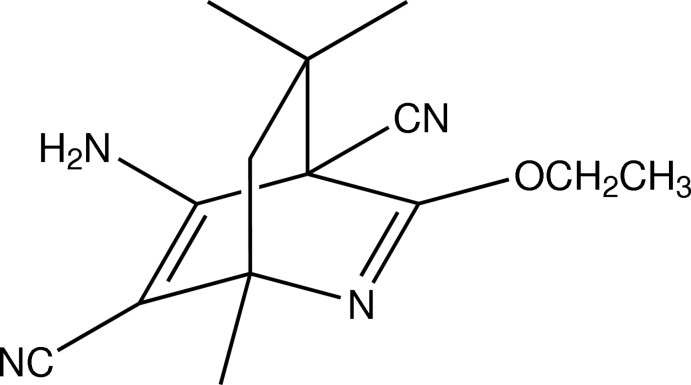


## Experimental

### 

#### Crystal data

C_14_H_18_N_4_O
*M* *_r_* = 258.32
Triclinic, 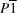

*a* = 9.1115 (1) Å
*b* = 12.4407 (2) Å
*c* = 13.4945 (2) Å
α = 62.945 (1)°
β = 85.382 (1)°
γ = 89.339 (1)°
*V* = 1357.30 (4) Å^3^
*Z* = 4
Mo *K*α radiation
μ = 0.08 mm^−1^
*T* = 100 K
0.37 × 0.15 × 0.09 mm

#### Data collection

Bruker APEXII CCD area-detector diffractometer
Absorption correction: multi-scan (*SADABS*; Bruker, 2009 ▶) *T*_min_ = 0.970, *T*_max_ = 0.993
23432 measured reflections
6248 independent reflections
5008 reflections with *I* > 2σ(*I*)
*R*_int_ = 0.041

#### Refinement

*R*[*F* ^2^ > 2σ(*F* ^2^)] = 0.047
*wR*(*F* ^2^) = 0.109
*S* = 1.05
6248 reflections
367 parameters
H atoms treated by a mixture of independent and constrained refinement
Δρ_max_ = 0.39 e Å^−3^
Δρ_min_ = −0.26 e Å^−3^

### 

Data collection: *APEX2* (Bruker, 2009 ▶); cell refinement: *SAINT* (Bruker, 2009 ▶); data reduction: *SAINT*; program(s) used to solve structure: *SHELXTL* (Sheldrick, 2008 ▶); program(s) used to refine structure: *SHELXTL*; molecular graphics: *SHELXTL*; software used to prepare material for publication: *SHELXTL* and *PLATON* (Spek, 2009 ▶).

## Supplementary Material

Crystal structure: contains datablock(s) global, I. DOI: 10.1107/S1600536812032990/rz2787sup1.cif

Structure factors: contains datablock(s) I. DOI: 10.1107/S1600536812032990/rz2787Isup2.hkl

Supplementary material file. DOI: 10.1107/S1600536812032990/rz2787Isup3.cml

Additional supplementary materials:  crystallographic information; 3D view; checkCIF report

## Figures and Tables

**Table 1:** Hydrogen-bond geometry (Å, °)

*D*—H⋯*A*	*D*—H	H⋯*A*	*D*⋯*A*	*D*—H⋯*A*
N2*A*—H1*NA*⋯O1*A* ^i^	0.92 (2)	2.57 (2)	3.4202 (17)	153.7 (17)
N2*A*—H2*NA*⋯N1*B* ^ii^	0.87 (2)	2.10 (2)	2.958 (2)	170 (2)
N2*B*—H1*NB*⋯N1*A* ^iii^	0.91 (2)	2.06 (2)	2.951 (2)	169 (2)
N2*B*—H2*NB*⋯O1*B* ^iv^	0.887 (19)	2.53 (2)	3.3524 (17)	154.4 (18)

Symmetry codes: (i) 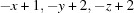; (ii) 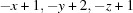; (iii) 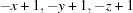; (iv) 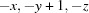.

**Fig. 1.**
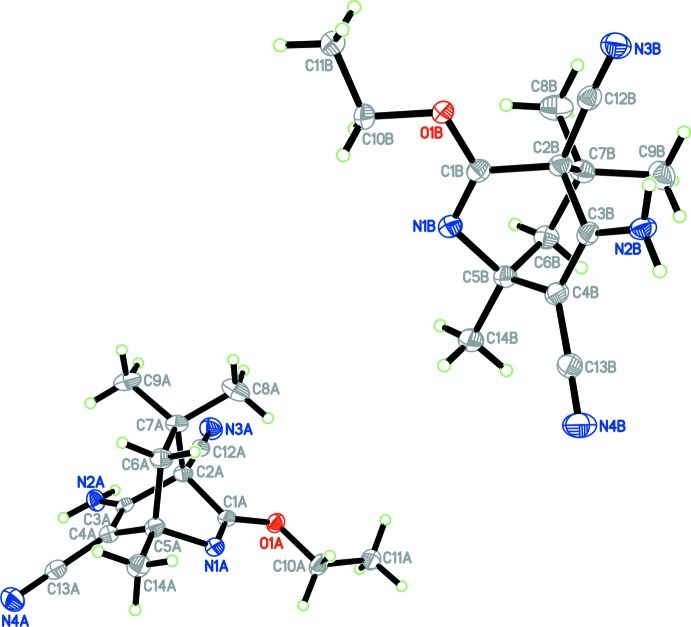
The molecular structure of the title compound, showing 40% probability displacement ellipsoids.

**Fig. 2.**
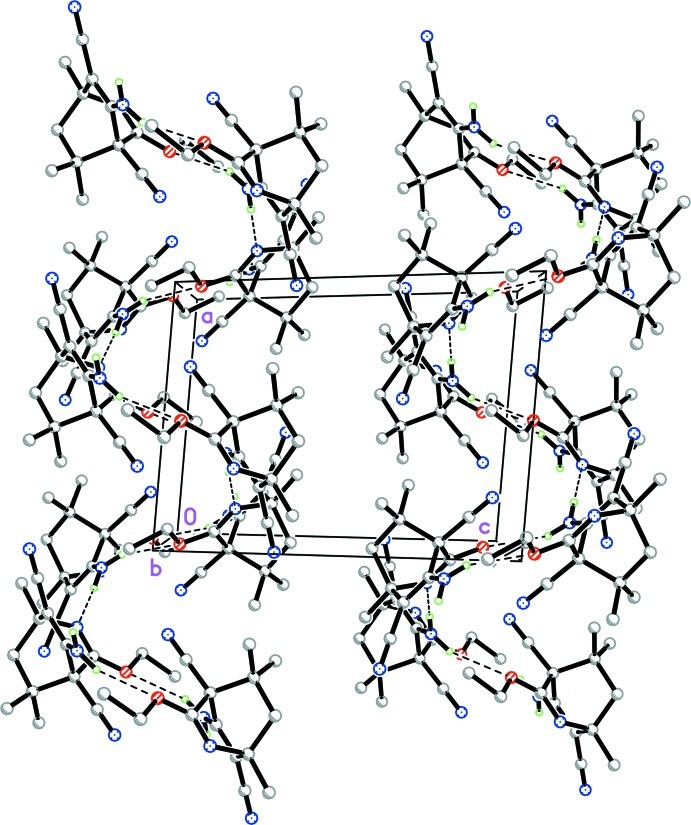
The crystal packing of the title compound viewed along the b axis, showing the two-dimensional networks. Only H atoms involved in hydrogen bonds (dashed lines) are shown.

## supplementary crystallographic information

### Comment

There are several reports showing that alkylidenemalononitriles can undergo self-condensation in the presence of alkoxide to give 2-azabicyclo[2.2.2]octa-2,5-diene derivatives (Igarashi *et al.*, 1987; Nakano & Igarashi, 1987; Nakano *et al.*, 1999). Herein we report the crystal structure of a new 2-azabicyclo[2.2.2]octa-2,5-diene derivative which was obtained from the self-condensation of malononitrile with acetone in the presence of sodium ethoxide.

The asymmetric of the title compound contains two crystallographic independent molecules *A* and *B* with similar conformation but differences in bond angles (Fig. 1). In both molecules *A* and *B*, the three six-membered rings are in boat conformation (Cremer & Pople, 1975). The molecules exist in the enamine form as indicated by the two H atoms attached to atom N2 and the C3 ═C4 is double bond [1.359 (1) Å in molecule *A* and 1.356 (2) Å in molecule *B*]. The angles around atom C1 indicate a *sp*_2_ hybridization [115.65(12 - 125.80 (13)° in molecule *A*; 115.55 (12) - 125.50 (13)° in molecule *B*]. The orientation of the ethoxy substituent can be indicated by the torsion angle C1–O1–C10–C11 = -174.53 (12)° in molecule *A* [-174.73 (12)° in molecule *B*]. The bond distances agree with the literature values (Allen *et al.*, 1987) and are comparable with those reported for a related structure (Nakano & Igarashi, 1987).

In the crystal packing (Fig. 2), the molecules are linked by intermolecular N—H···N and N—H···O hydrogen bonds (Table 1) into two dimensional networks parallel to the *ab* plane.

### Experimental

The title compound was obtained by the condensation reaction of malononitrile (1.5 mmol) with acetone (20 ml) in the presence of freshly prepared sodium ethoxide (1.0 mmol of sodium in 20 ml of ethanol). The mixture was continuously stirred at room temperature until a precipitate was formed. The resulting solid was filtered. Colourless block-shaped single crystals of the title compound suitable for X-ray structure determination were recrystalized from acetone/methanol (1:1 *v*/*v*) by the slow evaporation of the solvent at room temperature after several days.

### Refinement

Amino H atoms were located in a Fourier difference map and isotropically refined. The remaining H atoms were positioned geometrically and allowed to ride on their parent atoms, with d(C—H) = 0.99 Å for CH_2_ and 0.98 Å for CH_3_ atoms. The *U*_iso_ values were constrained to be 1.5*U*_eq_ of the carrier atom for methyl H atoms and 1.2*U*_eq_ for the remaining H atoms. A rotating group model was used for the methyl groups.

### Crystal data

**Table:**

C_14_H_18_N_4_O	*Z* = 4
*M**_r_* = 258.32	*F*(000) = 552
Triclinic, *P*1	*D*_x_ = 1.264 Mg m^−^^3^
Hall symbol: -P 1	Mo *K*α radiation, λ = 0.71073 Å
*a* = 9.1115 (1) Å	Cell parameters from 6248 reflections
*b* = 12.4407 (2) Å	θ = 1.8–27.6°
*c* = 13.4945 (2) Å	µ = 0.08 mm^−^^1^
α = 62.945 (1)°	*T* = 100 K
β = 85.382 (1)°	Block, colourles
γ = 89.339 (1)°	0.37 × 0.15 × 0.09 mm
*V* = 1357.30 (4) Å^3^	

### Data collection

**Table:**

Bruker APEXII CCD area-detector diffractometer	6248 independent reflections
Radiation source: sealed tube	5008 reflections with *I* > 2σ(*I*)
Graphite monochromator	*R*_int_ = 0.041
φ and ω scans	θ_max_ = 27.6°, θ_min_ = 1.7°
Absorption correction: multi-scan (*SADABS*; Bruker, 2009)	*h* = −11→11
*T*_min_ = 0.970, *T*_max_ = 0.993	*k* = −16→16
23432 measured reflections	*l* = −16→17

### Refinement

**Table:**

Refinement on *F*^2^	Primary atom site location: structure-invariant direct methods
Least-squares matrix: full	Secondary atom site location: difference Fourier map
*R*[*F*^2^ > 2σ(*F*^2^)] = 0.047	Hydrogen site location: inferred from neighbouring sites
*wR*(*F*^2^) = 0.109	H atoms treated by a mixture of independent and constrained refinement
*S* = 1.05	*w* = 1/[σ^2^(*F*_o_^2^) + (0.0415*P*)^2^ + 0.6407*P*] where *P* = (*F*_o_^2^ + 2*F*_c_^2^)/3
6248 reflections	(Δ/σ)_max_ < 0.001
367 parameters	Δρ_max_ = 0.39 e Å^−^^3^
0 restraints	Δρ_min_ = −0.26 e Å^−^^3^

### Special details

**Table:**

Experimental. The crystal was placed in the cold stream of an Oxford Cryosystems Cobra open-flow nitrogen cryostat (Cosier & Glazer, 1986) operating at 100.0 (1) K.
Geometry. All e.s.d.'s (except the e.s.d. in the dihedral angle between two l.s. planes) are estimated using the full covariance matrix. The cell e.s.d.'s are taken into account individually in the estimation of e.s.d.'s in distances, angles and torsion angles; correlations between e.s.d.'s in cell parameters are only used when they are defined by crystal symmetry. An approximate (isotropic) treatment of cell e.s.d.'s is used for estimating e.s.d.'s involving l.s. planes.
Refinement. Refinement of *F*^2^ against ALL reflections. The weighted *R*-factor *wR* and goodness of fit *S* are based on *F*^2^, conventional *R*-factors *R* are based on *F*, with *F* set to zero for negative *F*^2^. The threshold expression of *F*^2^ > σ(*F*^2^) is used only for calculating *R*-factors(gt) *etc*. and is not relevant to the choice of reflections for refinement. *R*-factors based on *F*^2^ are statistically about twice as large as those based on *F*, and *R*- factors based on ALL data will be even larger.

### Fractional atomic coordinates and isotropic or equivalent isotropic displacement parameters (Å^2^)

**Table:**

	*x*	*y*	*z*	*U*_iso_*/*U*_eq_	
O1A	0.51622 (11)	0.81541 (9)	0.93597 (8)	0.0146 (2)	
N1A	0.68788 (13)	0.88613 (11)	0.78197 (10)	0.0140 (3)	
N2A	0.64063 (15)	1.19295 (12)	0.82159 (11)	0.0161 (3)	
N3A	0.31531 (14)	1.04786 (12)	0.93804 (11)	0.0213 (3)	
N4A	1.00936 (15)	1.21629 (13)	0.65912 (12)	0.0235 (3)	
C1A	0.58254 (15)	0.90145 (13)	0.84112 (12)	0.0131 (3)	
C2A	0.51981 (15)	1.02696 (13)	0.80102 (12)	0.0134 (3)	
C3A	0.65055 (15)	1.11279 (13)	0.78038 (12)	0.0135 (3)	
C4A	0.76098 (15)	1.09834 (13)	0.71388 (12)	0.0144 (3)	
C5A	0.72908 (16)	1.00115 (13)	0.67865 (12)	0.0144 (3)	
C6A	0.58991 (16)	1.03710 (14)	0.61325 (12)	0.0162 (3)	
H6AA	0.5619	0.9721	0.5948	0.019*	
H6AB	0.6119	1.1115	0.5423	0.019*	
C7A	0.45923 (16)	1.05909 (14)	0.68268 (12)	0.0156 (3)	
C8A	0.32783 (17)	0.97506 (16)	0.70002 (14)	0.0235 (4)	
H8AA	0.2946	0.9912	0.6274	0.035*	
H8AB	0.2473	0.9893	0.7450	0.035*	
H8AC	0.3574	0.8909	0.7388	0.035*	
C9A	0.41209 (18)	1.19086 (15)	0.62827 (13)	0.0221 (3)	
H9AA	0.3778	1.2113	0.5549	0.033*	
H9AB	0.4962	1.2439	0.6192	0.033*	
H9AC	0.3322	1.2016	0.6758	0.033*	
C10A	0.57397 (17)	0.69428 (13)	0.97043 (13)	0.0179 (3)	
H10A	0.6771	0.6922	0.9893	0.021*	
H10B	0.5719	0.6716	0.9091	0.021*	
C11A	0.47834 (16)	0.60812 (14)	1.07080 (13)	0.0180 (3)	
H11A	0.5128	0.5258	1.0947	0.027*	
H11B	0.3762	0.6123	1.0516	0.027*	
H11C	0.4835	0.6300	1.1316	0.027*	
C12A	0.40365 (16)	1.03563 (13)	0.87895 (13)	0.0149 (3)	
C13A	0.89753 (16)	1.16370 (13)	0.68368 (12)	0.0162 (3)	
C14A	0.85776 (16)	0.97961 (14)	0.61147 (13)	0.0185 (3)	
H14A	0.9435	0.9561	0.6555	0.028*	
H14B	0.8819	1.0539	0.5423	0.028*	
H14C	0.8309	0.9150	0.5933	0.028*	
O1B	0.00455 (11)	0.68930 (9)	0.05704 (8)	0.0147 (2)	
N1B	0.14158 (13)	0.61679 (11)	0.21120 (10)	0.0145 (3)	
N2B	0.09705 (14)	0.30548 (12)	0.17838 (11)	0.0157 (3)	
N3B	−0.19771 (14)	0.45877 (12)	0.05722 (11)	0.0206 (3)	
N4B	0.43461 (15)	0.28183 (13)	0.33393 (12)	0.0240 (3)	
C1B	0.04928 (15)	0.60238 (13)	0.15241 (12)	0.0135 (3)	
C2B	−0.02378 (15)	0.47844 (13)	0.19317 (12)	0.0135 (3)	
C3B	0.10078 (15)	0.38876 (13)	0.21586 (12)	0.0135 (3)	
C4B	0.19768 (16)	0.40264 (13)	0.28067 (12)	0.0145 (3)	
C5B	0.15887 (16)	0.50179 (13)	0.31466 (12)	0.0145 (3)	
C6B	0.00466 (16)	0.46929 (14)	0.38005 (12)	0.0157 (3)	
H6BA	0.0101	0.3944	0.4509	0.019*	
H6BB	−0.0264	0.5349	0.3987	0.019*	
C7B	−0.11103 (16)	0.45074 (14)	0.31062 (12)	0.0155 (3)	
C8B	−0.23621 (17)	0.53982 (16)	0.29022 (14)	0.0224 (3)	
H8BA	−0.2892	0.5244	0.3618	0.034*	
H8BB	−0.3044	0.5295	0.2424	0.034*	
H8BC	−0.1952	0.6226	0.2534	0.034*	
C9B	−0.17408 (17)	0.32157 (15)	0.36637 (13)	0.0216 (3)	
H9BA	−0.2243	0.3039	0.4393	0.032*	
H9BB	−0.0939	0.2653	0.3764	0.032*	
H9BC	−0.2444	0.3126	0.3192	0.032*	
C10B	0.06840 (17)	0.80957 (13)	0.02335 (13)	0.0177 (3)	
H10C	0.1761	0.8104	0.0056	0.021*	
H10D	0.0505	0.8322	0.0847	0.021*	
C11B	−0.00375 (16)	0.89715 (13)	−0.07808 (13)	0.0179 (3)	
H11D	0.0367	0.9787	−0.1024	0.027*	
H11E	−0.1102	0.8955	−0.0596	0.027*	
H11F	0.0152	0.8742	−0.1384	0.027*	
C12B	−0.12256 (16)	0.47068 (13)	0.11584 (12)	0.0149 (3)	
C13B	0.32758 (16)	0.33519 (14)	0.31065 (12)	0.0162 (3)	
C14B	0.27220 (16)	0.52006 (14)	0.38244 (13)	0.0179 (3)	
H14D	0.3683	0.5408	0.3391	0.027*	
H14E	0.2793	0.4455	0.4519	0.027*	
H14F	0.2423	0.5857	0.4001	0.027*	
H1NA	0.573 (2)	1.1790 (18)	0.8807 (18)	0.035 (5)*	
H2NA	0.713 (2)	1.2426 (18)	0.8112 (15)	0.024 (5)*	
H1NB	0.172 (2)	0.2538 (19)	0.1900 (17)	0.032 (5)*	
H2NB	0.046 (2)	0.3204 (17)	0.1204 (16)	0.024 (5)*	

### Atomic displacement parameters (Å^2^)

**Table:**

	*U*^11^	*U*^22^	*U*^33^	*U*^12^	*U*^13^	*U*^23^
O1A	0.0143 (5)	0.0113 (5)	0.0163 (5)	0.0015 (4)	0.0003 (4)	−0.0050 (4)
N1A	0.0134 (6)	0.0122 (6)	0.0157 (6)	0.0012 (5)	−0.0026 (5)	−0.0057 (5)
N2A	0.0158 (6)	0.0149 (6)	0.0194 (7)	−0.0016 (5)	0.0001 (5)	−0.0097 (5)
N3A	0.0178 (6)	0.0224 (7)	0.0240 (7)	0.0011 (5)	−0.0002 (6)	−0.0111 (6)
N4A	0.0165 (7)	0.0224 (7)	0.0301 (8)	−0.0005 (6)	0.0002 (6)	−0.0111 (6)
C1A	0.0119 (6)	0.0128 (7)	0.0146 (7)	0.0010 (5)	−0.0043 (5)	−0.0058 (6)
C2A	0.0111 (6)	0.0134 (7)	0.0155 (7)	0.0021 (5)	−0.0014 (5)	−0.0065 (6)
C3A	0.0132 (7)	0.0106 (7)	0.0140 (7)	0.0030 (5)	−0.0051 (5)	−0.0028 (6)
C4A	0.0128 (7)	0.0130 (7)	0.0158 (7)	0.0016 (5)	−0.0026 (5)	−0.0050 (6)
C5A	0.0139 (7)	0.0145 (7)	0.0141 (7)	0.0023 (5)	−0.0014 (5)	−0.0057 (6)
C6A	0.0160 (7)	0.0182 (7)	0.0153 (7)	0.0017 (6)	−0.0023 (6)	−0.0082 (6)
C7A	0.0137 (7)	0.0186 (7)	0.0152 (7)	0.0029 (6)	−0.0042 (6)	−0.0077 (6)
C8A	0.0159 (7)	0.0334 (9)	0.0247 (9)	−0.0010 (7)	−0.0039 (6)	−0.0160 (7)
C9A	0.0204 (8)	0.0237 (8)	0.0201 (8)	0.0096 (6)	−0.0067 (6)	−0.0077 (7)
C10A	0.0191 (7)	0.0116 (7)	0.0208 (8)	0.0033 (6)	0.0003 (6)	−0.0059 (6)
C11A	0.0170 (7)	0.0151 (7)	0.0194 (8)	0.0005 (6)	−0.0005 (6)	−0.0058 (6)
C12A	0.0132 (7)	0.0129 (7)	0.0187 (7)	0.0010 (5)	−0.0044 (6)	−0.0068 (6)
C13A	0.0169 (7)	0.0148 (7)	0.0155 (7)	0.0041 (6)	−0.0026 (6)	−0.0054 (6)
C14A	0.0179 (7)	0.0174 (8)	0.0192 (8)	0.0028 (6)	0.0013 (6)	−0.0081 (6)
O1B	0.0157 (5)	0.0113 (5)	0.0160 (5)	0.0008 (4)	−0.0043 (4)	−0.0048 (4)
N1B	0.0142 (6)	0.0141 (6)	0.0149 (6)	0.0019 (5)	−0.0019 (5)	−0.0062 (5)
N2B	0.0155 (6)	0.0155 (6)	0.0184 (7)	0.0054 (5)	−0.0057 (5)	−0.0091 (5)
N3B	0.0175 (6)	0.0222 (7)	0.0239 (7)	0.0031 (5)	−0.0054 (6)	−0.0117 (6)
N4B	0.0207 (7)	0.0266 (8)	0.0293 (8)	0.0076 (6)	−0.0099 (6)	−0.0158 (6)
C1B	0.0133 (7)	0.0133 (7)	0.0140 (7)	0.0024 (5)	−0.0003 (5)	−0.0065 (6)
C2B	0.0122 (6)	0.0136 (7)	0.0154 (7)	0.0024 (5)	−0.0022 (5)	−0.0071 (6)
C3B	0.0129 (7)	0.0114 (7)	0.0136 (7)	0.0007 (5)	0.0004 (5)	−0.0037 (6)
C4B	0.0138 (7)	0.0134 (7)	0.0144 (7)	0.0018 (5)	−0.0018 (5)	−0.0048 (6)
C5B	0.0149 (7)	0.0141 (7)	0.0147 (7)	0.0012 (5)	−0.0034 (5)	−0.0064 (6)
C6B	0.0158 (7)	0.0182 (7)	0.0132 (7)	0.0007 (6)	−0.0012 (6)	−0.0074 (6)
C7B	0.0134 (7)	0.0187 (7)	0.0145 (7)	0.0001 (6)	−0.0003 (6)	−0.0078 (6)
C8B	0.0172 (7)	0.0314 (9)	0.0217 (8)	0.0076 (7)	−0.0017 (6)	−0.0150 (7)
C9B	0.0194 (8)	0.0230 (8)	0.0193 (8)	−0.0045 (6)	0.0007 (6)	−0.0072 (7)
C10B	0.0203 (7)	0.0115 (7)	0.0208 (8)	−0.0013 (6)	−0.0057 (6)	−0.0063 (6)
C11B	0.0169 (7)	0.0148 (7)	0.0203 (8)	0.0012 (6)	−0.0037 (6)	−0.0061 (6)
C12B	0.0136 (7)	0.0132 (7)	0.0165 (7)	0.0022 (5)	−0.0001 (6)	−0.0058 (6)
C13B	0.0176 (7)	0.0162 (7)	0.0155 (7)	0.0005 (6)	−0.0037 (6)	−0.0075 (6)
C14B	0.0178 (7)	0.0185 (8)	0.0180 (8)	0.0010 (6)	−0.0054 (6)	−0.0083 (6)

### Geometric parameters (Å, º)

**Table:**

O1A—C1A	1.3391 (17)	O1B—C1B	1.3417 (17)
O1A—C10A	1.4654 (17)	O1B—C10B	1.4638 (17)
N1A—C1A	1.2671 (18)	N1B—C1B	1.2684 (19)
N1A—C5A	1.4986 (18)	N1B—C5B	1.4947 (18)
N2A—C3A	1.3440 (19)	N2B—C3B	1.3456 (19)
N2A—H1NA	0.92 (2)	N2B—H1NB	0.91 (2)
N2A—H2NA	0.87 (2)	N2B—H2NB	0.89 (2)
N3A—C12A	1.145 (2)	N3B—C12B	1.146 (2)
N4A—C13A	1.158 (2)	N4B—C13B	1.155 (2)
C1A—C2A	1.5226 (19)	C1B—C2B	1.522 (2)
C2A—C12A	1.469 (2)	C2B—C12B	1.467 (2)
C2A—C3A	1.530 (2)	C2B—C3B	1.5305 (19)
C2A—C7A	1.603 (2)	C2B—C7B	1.6029 (19)
C3A—C4A	1.359 (2)	C3B—C4B	1.356 (2)
C4A—C13A	1.420 (2)	C4B—C13B	1.419 (2)
C4A—C5A	1.525 (2)	C4B—C5B	1.528 (2)
C5A—C14A	1.5202 (19)	C5B—C14B	1.517 (2)
C5A—C6A	1.548 (2)	C5B—C6B	1.551 (2)
C6A—C7A	1.554 (2)	C6B—C7B	1.550 (2)
C6A—H6AA	0.9900	C6B—H6BA	0.9900
C6A—H6AB	0.9900	C6B—H6BB	0.9900
C7A—C8A	1.531 (2)	C7B—C9B	1.526 (2)
C7A—C9A	1.533 (2)	C7B—C8B	1.533 (2)
C8A—H8AA	0.9800	C8B—H8BA	0.9800
C8A—H8AB	0.9800	C8B—H8BB	0.9800
C8A—H8AC	0.9800	C8B—H8BC	0.9800
C9A—H9AA	0.9800	C9B—H9BA	0.9800
C9A—H9AB	0.9800	C9B—H9BB	0.9800
C9A—H9AC	0.9800	C9B—H9BC	0.9800
C10A—C11A	1.501 (2)	C10B—C11B	1.505 (2)
C10A—H10A	0.9900	C10B—H10C	0.9900
C10A—H10B	0.9900	C10B—H10D	0.9900
C11A—H11A	0.9800	C11B—H11D	0.9800
C11A—H11B	0.9800	C11B—H11E	0.9800
C11A—H11C	0.9800	C11B—H11F	0.9800
C14A—H14A	0.9800	C14B—H14D	0.9800
C14A—H14B	0.9800	C14B—H14E	0.9800
C14A—H14C	0.9800	C14B—H14F	0.9800
			
C1A—O1A—C10A	114.77 (11)	C1B—O1B—C10B	114.72 (11)
C1A—N1A—C5A	111.14 (12)	C1B—N1B—C5B	110.77 (12)
C3A—N2A—H1NA	119.3 (13)	C3B—N2B—H1NB	119.6 (13)
C3A—N2A—H2NA	121.5 (12)	C3B—N2B—H2NB	118.6 (12)
H1NA—N2A—H2NA	115.6 (18)	H1NB—N2B—H2NB	116.8 (17)
N1A—C1A—O1A	125.80 (13)	N1B—C1B—O1B	125.50 (13)
N1A—C1A—C2A	118.51 (13)	N1B—C1B—C2B	118.90 (13)
O1A—C1A—C2A	115.65 (12)	O1B—C1B—C2B	115.55 (12)
C12A—C2A—C1A	113.79 (12)	C12B—C2B—C1B	114.18 (12)
C12A—C2A—C3A	111.62 (12)	C12B—C2B—C3B	111.54 (12)
C1A—C2A—C3A	106.25 (11)	C1B—C2B—C3B	106.50 (11)
C12A—C2A—C7A	111.29 (11)	C12B—C2B—C7B	111.31 (11)
C1A—C2A—C7A	105.59 (11)	C1B—C2B—C7B	105.00 (11)
C3A—C2A—C7A	107.90 (11)	C3B—C2B—C7B	107.89 (11)
N2A—C3A—C4A	129.35 (14)	N2B—C3B—C4B	129.14 (14)
N2A—C3A—C2A	119.54 (13)	N2B—C3B—C2B	119.52 (13)
C4A—C3A—C2A	110.99 (13)	C4B—C3B—C2B	111.18 (13)
C3A—C4A—C13A	123.22 (14)	C3B—C4B—C13B	123.34 (14)
C3A—C4A—C5A	114.30 (13)	C3B—C4B—C5B	114.15 (13)
C13A—C4A—C5A	122.42 (13)	C13B—C4B—C5B	122.45 (13)
N1A—C5A—C14A	109.48 (12)	N1B—C5B—C14B	109.98 (12)
N1A—C5A—C4A	108.12 (11)	N1B—C5B—C4B	108.41 (12)
C14A—C5A—C4A	113.69 (12)	C14B—C5B—C4B	113.42 (12)
N1A—C5A—C6A	105.84 (11)	N1B—C5B—C6B	105.86 (11)
C14A—C5A—C6A	111.55 (12)	C14B—C5B—C6B	111.21 (12)
C4A—C5A—C6A	107.80 (12)	C4B—C5B—C6B	107.64 (12)
C5A—C6A—C7A	111.07 (12)	C7B—C6B—C5B	111.13 (12)
C5A—C6A—H6AA	109.4	C7B—C6B—H6BA	109.4
C7A—C6A—H6AA	109.4	C5B—C6B—H6BA	109.4
C5A—C6A—H6AB	109.4	C7B—C6B—H6BB	109.4
C7A—C6A—H6AB	109.4	C5B—C6B—H6BB	109.4
H6AA—C6A—H6AB	108.0	H6BA—C6B—H6BB	108.0
C8A—C7A—C9A	110.00 (13)	C9B—C7B—C8B	109.86 (12)
C8A—C7A—C6A	110.68 (12)	C9B—C7B—C6B	111.77 (13)
C9A—C7A—C6A	112.01 (12)	C8B—C7B—C6B	111.21 (13)
C8A—C7A—C2A	109.42 (12)	C9B—C7B—C2B	109.64 (12)
C9A—C7A—C2A	109.39 (12)	C8B—C7B—C2B	108.93 (12)
C6A—C7A—C2A	105.21 (11)	C6B—C7B—C2B	105.29 (11)
C7A—C8A—H8AA	109.5	C7B—C8B—H8BA	109.5
C7A—C8A—H8AB	109.5	C7B—C8B—H8BB	109.5
H8AA—C8A—H8AB	109.5	H8BA—C8B—H8BB	109.5
C7A—C8A—H8AC	109.5	C7B—C8B—H8BC	109.5
H8AA—C8A—H8AC	109.5	H8BA—C8B—H8BC	109.5
H8AB—C8A—H8AC	109.5	H8BB—C8B—H8BC	109.5
C7A—C9A—H9AA	109.5	C7B—C9B—H9BA	109.5
C7A—C9A—H9AB	109.5	C7B—C9B—H9BB	109.5
H9AA—C9A—H9AB	109.5	H9BA—C9B—H9BB	109.5
C7A—C9A—H9AC	109.5	C7B—C9B—H9BC	109.5
H9AA—C9A—H9AC	109.5	H9BA—C9B—H9BC	109.5
H9AB—C9A—H9AC	109.5	H9BB—C9B—H9BC	109.5
O1A—C10A—C11A	107.60 (11)	O1B—C10B—C11B	107.68 (12)
O1A—C10A—H10A	110.2	O1B—C10B—H10C	110.2
C11A—C10A—H10A	110.2	C11B—C10B—H10C	110.2
O1A—C10A—H10B	110.2	O1B—C10B—H10D	110.2
C11A—C10A—H10B	110.2	C11B—C10B—H10D	110.2
H10A—C10A—H10B	108.5	H10C—C10B—H10D	108.5
C10A—C11A—H11A	109.5	C10B—C11B—H11D	109.5
C10A—C11A—H11B	109.5	C10B—C11B—H11E	109.5
H11A—C11A—H11B	109.5	H11D—C11B—H11E	109.5
C10A—C11A—H11C	109.5	C10B—C11B—H11F	109.5
H11A—C11A—H11C	109.5	H11D—C11B—H11F	109.5
H11B—C11A—H11C	109.5	H11E—C11B—H11F	109.5
N3A—C12A—C2A	176.79 (15)	N3B—C12B—C2B	176.58 (15)
N4A—C13A—C4A	179.54 (16)	N4B—C13B—C4B	178.95 (18)
C5A—C14A—H14A	109.5	C5B—C14B—H14D	109.5
C5A—C14A—H14B	109.5	C5B—C14B—H14E	109.5
H14A—C14A—H14B	109.5	H14D—C14B—H14E	109.5
C5A—C14A—H14C	109.5	C5B—C14B—H14F	109.5
H14A—C14A—H14C	109.5	H14D—C14B—H14F	109.5
H14B—C14A—H14C	109.5	H14E—C14B—H14F	109.5
			
C5A—N1A—C1A—O1A	177.02 (13)	C5B—N1B—C1B—O1B	177.00 (12)
C5A—N1A—C1A—C2A	−0.75 (18)	C5B—N1B—C1B—C2B	−0.39 (17)
C10A—O1A—C1A—N1A	−0.4 (2)	C10B—O1B—C1B—N1B	−1.5 (2)
C10A—O1A—C1A—C2A	177.40 (12)	C10B—O1B—C1B—C2B	175.93 (11)
N1A—C1A—C2A—C12A	−176.91 (13)	N1B—C1B—C2B—C12B	−176.98 (12)
O1A—C1A—C2A—C12A	5.09 (18)	O1B—C1B—C2B—C12B	5.38 (17)
N1A—C1A—C2A—C3A	−53.69 (17)	N1B—C1B—C2B—C3B	−53.43 (16)
O1A—C1A—C2A—C3A	128.31 (13)	O1B—C1B—C2B—C3B	128.93 (13)
N1A—C1A—C2A—C7A	60.75 (16)	N1B—C1B—C2B—C7B	60.84 (16)
O1A—C1A—C2A—C7A	−117.25 (13)	O1B—C1B—C2B—C7B	−116.80 (13)
C12A—C2A—C3A—N2A	−7.51 (19)	C12B—C2B—C3B—N2B	−8.73 (18)
C1A—C2A—C3A—N2A	−132.08 (13)	C1B—C2B—C3B—N2B	−133.91 (13)
C7A—C2A—C3A—N2A	115.07 (14)	C7B—C2B—C3B—N2B	113.81 (14)
C12A—C2A—C3A—C4A	176.11 (12)	C12B—C2B—C3B—C4B	175.44 (13)
C1A—C2A—C3A—C4A	51.54 (16)	C1B—C2B—C3B—C4B	50.26 (15)
C7A—C2A—C3A—C4A	−61.31 (15)	C7B—C2B—C3B—C4B	−62.02 (15)
N2A—C3A—C4A—C13A	6.0 (2)	N2B—C3B—C4B—C13B	7.6 (2)
C2A—C3A—C4A—C13A	−178.06 (13)	C2B—C3B—C4B—C13B	−177.03 (13)
N2A—C3A—C4A—C5A	−176.84 (14)	N2B—C3B—C4B—C5B	−175.05 (14)
C2A—C3A—C4A—C5A	−0.91 (17)	C2B—C3B—C4B—C5B	0.27 (17)
C1A—N1A—C5A—C14A	178.74 (13)	C1B—N1B—C5B—C14B	178.68 (12)
C1A—N1A—C5A—C4A	54.39 (15)	C1B—N1B—C5B—C4B	54.17 (15)
C1A—N1A—C5A—C6A	−60.89 (15)	C1B—N1B—C5B—C6B	−61.09 (15)
C3A—C4A—C5A—N1A	−53.95 (16)	C3B—C4B—C5B—N1B	−54.80 (16)
C13A—C4A—C5A—N1A	123.23 (14)	C13B—C4B—C5B—N1B	122.53 (14)
C3A—C4A—C5A—C14A	−175.75 (13)	C3B—C4B—C5B—C14B	−177.24 (13)
C13A—C4A—C5A—C14A	1.4 (2)	C13B—C4B—C5B—C14B	0.1 (2)
C3A—C4A—C5A—C6A	60.05 (16)	C3B—C4B—C5B—C6B	59.29 (16)
C13A—C4A—C5A—C6A	−122.77 (14)	C13B—C4B—C5B—C6B	−123.38 (14)
N1A—C5A—C6A—C7A	61.10 (15)	N1B—C5B—C6B—C7B	60.54 (15)
C14A—C5A—C6A—C7A	−179.90 (12)	C14B—C5B—C6B—C7B	179.96 (12)
C4A—C5A—C6A—C7A	−54.41 (15)	C4B—C5B—C6B—C7B	−55.24 (15)
C5A—C6A—C7A—C8A	−121.52 (14)	C5B—C6B—C7B—C9B	116.77 (13)
C5A—C6A—C7A—C9A	115.33 (14)	C5B—C6B—C7B—C8B	−120.04 (13)
C5A—C6A—C7A—C2A	−3.42 (16)	C5B—C6B—C7B—C2B	−2.20 (16)
C12A—C2A—C7A—C8A	−57.10 (16)	C12B—C2B—C7B—C9B	62.71 (15)
C1A—C2A—C7A—C8A	66.83 (14)	C1B—C2B—C7B—C9B	−173.27 (12)
C3A—C2A—C7A—C8A	−179.88 (12)	C3B—C2B—C7B—C9B	−59.97 (15)
C12A—C2A—C7A—C9A	63.47 (15)	C12B—C2B—C7B—C8B	−57.54 (16)
C1A—C2A—C7A—C9A	−172.60 (12)	C1B—C2B—C7B—C8B	66.48 (14)
C3A—C2A—C7A—C9A	−59.31 (14)	C3B—C2B—C7B—C8B	179.78 (12)
C12A—C2A—C7A—C6A	−176.04 (12)	C12B—C2B—C7B—C6B	−176.91 (12)
C1A—C2A—C7A—C6A	−52.11 (14)	C1B—C2B—C7B—C6B	−52.88 (14)
C3A—C2A—C7A—C6A	61.18 (14)	C3B—C2B—C7B—C6B	60.41 (14)
C1A—O1A—C10A—C11A	−174.53 (12)	C1B—O1B—C10B—C11B	−174.73 (12)

### Hydrogen-bond geometry (Å, º)

**Table:**

*D*—H···*A*	*D*—H	H···*A*	*D*···*A*	*D*—H···*A*
N2*A*—H1*NA*···O1*A*^i^	0.92 (2)	2.57 (2)	3.4202 (17)	153.7 (17)
N2*A*—H2*NA*···N1*B*^ii^	0.87 (2)	2.10 (2)	2.958 (2)	170 (2)
N2*B*—H1*NB*···N1*A*^iii^	0.91 (2)	2.06 (2)	2.951 (2)	169 (2)
N2*B*—H2*NB*···O1*B*^iv^	0.887 (19)	2.53 (2)	3.3524 (17)	154.4 (18)

Symmetry codes: (i) −*x*+1, −*y*+2, −*z*+2; (ii) −*x*+1, −*y*+2, −*z*+1; (iii) −*x*+1, −*y*+1, −*z*+1; (iv) −*x*, −*y*+1, −*z*.
